# Identification of Three lncRNAs as Potential Predictive Biomarkers of Lung Adenocarcinoma

**DOI:** 10.1155/2020/7573689

**Published:** 2020-02-19

**Authors:** Donghui Jin, Yuxuan Song, Yuan Chen, Peng Zhang

**Affiliations:** ^1^Cardiovascular Thoracic Surgery Department, Tianjin Medical University General Hospital, 154 Anshan Road, Heping District, Tianjin 300052, China; ^2^Department of Urology, Tianjin Medical University General Hospital, 154 Anshan Road, Heping District, Tianjin 300052, China

## Abstract

**Background:**

Lung cancer is the most common cancer and the most common cause of cancer-related death worldwide. However, the molecular mechanism of its development is unclear. It is imperative to identify more novel biomarkers.

**Methods:**

Two datasets (GSE70880 and GSE113852) were downloaded from the Gene Expression Omnibus (GEO) database and used to identify the differentially expressed genes (DEGs) between lung cancer tissues and normal tissues. Then, we constructed a competing endogenous RNA (ceRNA) network and a protein-protein interaction (PPI) network and performed gene ontology (GO) analysis, Kyoto Encyclopedia of Genes and Genomes (KEGG) pathway analysis, and survival analyses to identify potential biomarkers that are related to the diagnosis and prognosis of lung cancer.

**Results:**

A total of 41 lncRNAs and 805 mRNAs were differentially expressed in lung cancer. The ceRNA network contained four lncRNAs (CLDN10-AS1, SFTA1P, SRGAP3-AS2, and ADAMTS9-AS2), 21 miRNAs, and 48 mRNAs. Functional analyses revealed that the genes in the ceRNA network were mainly enriched in cell migration, transmembrane receptor, and protein kinase activity. mRNAs DLGAP5, E2F7, MCM7, RACGAP1, and RRM2 had the highest connectivity in the PPI network. Immunohistochemistry (IHC) demonstrated that mRNAs DLGAP5, MCM7, RACGAP1, and RRM2 were upregulated in lung adenocarcinoma (LUAD). Survival analyses showed that lncRNAs CLDN10-AS1, SFTA1P, and ADAMTS9-AS2 were associated with the prognosis of LUAD.

**Conclusion:**

lncRNAs CLDN10-AS1, SFTA1P, and ADAMTS9-AS2 might be the biomarkers of LUAD. For the first time, we confirmed the important role of lncRNA CLDN10-AS1 in LUAD.

## 1. Introduction

Lung cancer has the highest incidence of all cancers (11.6% of the total cases) and the highest death rate (18.4% of the total cancer deaths) [1]. It contains two main types, small cell (SCLC, 15% cases) and non-small-cell lung cancer (NSCLC, 85% cases). Lung adenocarcinoma (LUAD) and squamous cell carcinoma (LUSC) are the main histological subtypes of NSCLC [2]. The underlying biological and molecular mechanisms of lung cancer are gradually being understood over the past decades [3]. The molecular biomarkers may improve the early lung cancer detection [4]. Furthermore, traditional chemotherapy that is based on histopathology is replaced by the individualized precision treatment which is based on carcinogenic factors [5].

Protein-coding genes are widely studied in lung cancer over the past decades, but the human genome transcribes less than 2% of the protein-coding genes. 85% are noncoding RNAs, including long noncoding RNAs (lncRNAs) [6]. lncRNAs account for a large part of the human genome [7], and they were once considered to be the insignificant “noise” in genome's repertoire of non-protein-coding transcripts. However, recent studies have revealed the roles of lncRNAs in many biological processes, including transcriptional regulation and cell differentiation [8,9]. It is well recognized that dysregulation of lncRNAs plays an important role in cancer, including lung cancer [10,11]. Nevertheless, a large number of lncRNAs are still unexplored [12]. Therefore, it is imperative to recognize more lncRNAs as biomarkers of lung cancer for better diagnosis, therapy, and prediction of the prognosis.

This study aimed to explore more biomarkers of lung cancer via integrated bioinformatics analysis. Gene Expression Omnibus (GEO) is a NCBI's publicly available genomics database (https://www.ncbi.nlm.nih.gov/gds/), which provides us a large amount of genomic data about lung cancer. Two datasets (GSE70880 and GSE113852) were downloaded from GEO and used to identify the differentially expressed genes (DEGs) between lung cancer tissues and normal tissues. Then, we constructed a competing endogenous RNA (ceRNA) network and a protein-protein interaction (PPI) network and performed gene ontology (GO) analysis, Kyoto Encyclopedia of Genes and Genomes (KEGG) pathway analysis, and survival analyses to identify potential biomarkers that are related to the diagnosis and prognosis of lung cancer. We verified the expression differences and measured the diagnostic roles of lncRNAs through The Cancer Genome Atlas (TCGA) database (https://portal.gdc.cancer.gov/).

## 2. Materials and Methods

### 2.1. Gene Expression Microarray Datasets

We downloaded gene expression microarray datasets GSE70880 and GSE113852 from the GEO database. The criteria they met were as follows: (1) the studies were about lung cancer, (2) tissue samples included the tumor and corresponding adjacent tissues or normal tissues, (3) the number of total samples was not less than 40, and (4) the datasets must include lncRNAs and mRNAs.

### 2.2. Integrated Analysis of Microarray Datasets

We merged the two datasets not only to increase the sample size but also to facilitate subsequent analyses. Based on different platforms, days, environments, and people, the samples had heterogeneity and potential variables, which might lead to a bias. Therefore, we batch-normalized the merged dataset by suing limma and sva packages in software R (v3.6.1). Next, we performed gene differential analysis between tumor and normal tissues by limma package. |LogFC| > 1 and adjusted *P* value <0.05 (the correction for *P* value was done by Benjamini–Hochberg method) were considered statistically significant for the DEGs.

### 2.3. Construction of ceRNA Network

In order to find out the competing endogenous regulating network mediated by lncRNAs and miRNAs, the ceRNA network was constructed. CeRNA networks link the functions of protein-coding mRNAs with that of noncoding RNAs such as microRNA, long noncoding RNA, pseudogenic RNA, and circular RNA [12]. The construction of the ceRNA network included two steps. (1) Interactions between differentially expressed lncRNAs (DElncRNAs) and miRNAs were predicted by the miRcode database (http://www.mircode.org/) [13]. lncRNAs could competitively bind to the shared miRNAs. The upregulation and downregulation of one lncRNA result in more and less sequestrated copies of shared miRNAs, respectively [14]. (2) miRNA-mRNA interactions were predicted by miRTarBase (http://mirtarbase.mbc.nctu.edu.tw/) [15], TargetScan (http://www.targetscan.org/) [16], and miRDB (http://www.mirdb.org/miRDB/) [17]. The interactions should match all the three databases. We selected the mRNAs that were differentially expressed in our study from the target mRNAs of miRNAs for the construction of the ceRNA network. We used the software Cytoscape (v3.7.1) to visualize the network.

### 2.4. Functional Enrichment Analysis and PPI Network

Functional enrichment analysis includes GO and KEGG analysis. Genes in the ceRNA network were analyzed by GO and KEGG analysis by package clusterprofiler in software R. In GO analysis, functions were divided into biological processes (BP), molecular functions (MF), and cellular component (CC). PPI network was constructed by using STRING (http://string‐db.org/cgi/input.pl).

### 2.5. Online Survival Analyses in LUAD and LUSC

To confirm whether lncRNA is associated with the survival of LUAD and LUSC was one of our research contents. Due to lack of clinical information, we performed Kaplan–Meier (KM) analyses of lncRNAs in the ceRNA network and genes in the PPI network through the Kaplan–Meier Plotter (http://kmplot.com/analysis/), respectively [18]. We only performed the survival analyses of these lncRNAs and mRNAs in LUAD and LUSC. Log rank *P* < 0.05 was considered statistically significant. Genes related to the prognosis of LUAD or LUSC would be considered as the hub genes.

### 2.6. The Differences of Expression Levels and Diagnostic Roles of lncRNAs Selected from ceRNA Network

To confirm whether the expression levels of lncRNAs in the ceRNA network were different between normal and tumor tissues, we downloaded the gene expression profile from TCGA database and performed gene differential analysis of lncRNAs selected from the ceRNA network. *P* < 0.05 means there is a statistical difference. In order to measure the diagnostic values of these lncRNAs, we used the data from TCGA database to perform receiver operating characteristic (ROC) curves by GraphPad Prism 7.0, and the values of cutoff, specificity, sensitivity, and area under the curve (AUC) were also calculated. The cutoff value is the best expression of a lncRNA for the diagnosis of lung cancer. *P* < 0.05 indicated significant difference.

## 3. Results

### 3.1. Gene Expression Profile Data

**Figure 1:**
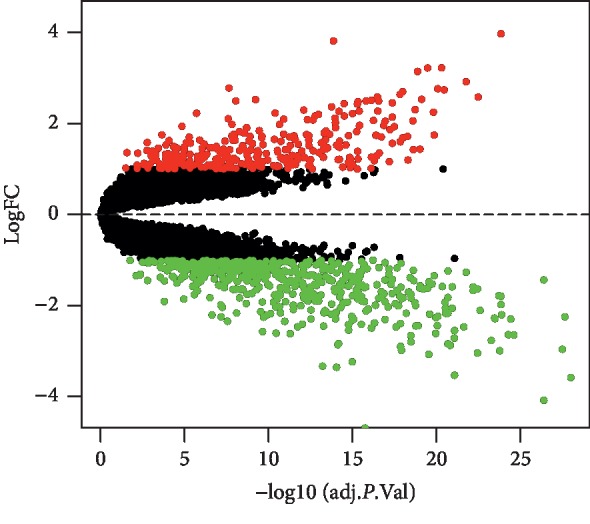
Volcano plot of differentially expressed genes in the merged dataset. Red dots represent genes upregulation and based on adjusted *P* < 0.05 and logFC > 1; green dots represent genes downregulation and based on adjusted *P* < 0.05 and logFC < −1; black dots represent genes being not significantly differentially expressed between tumor tissues and normal tissues. FC = fold change.

**Figure 2:**
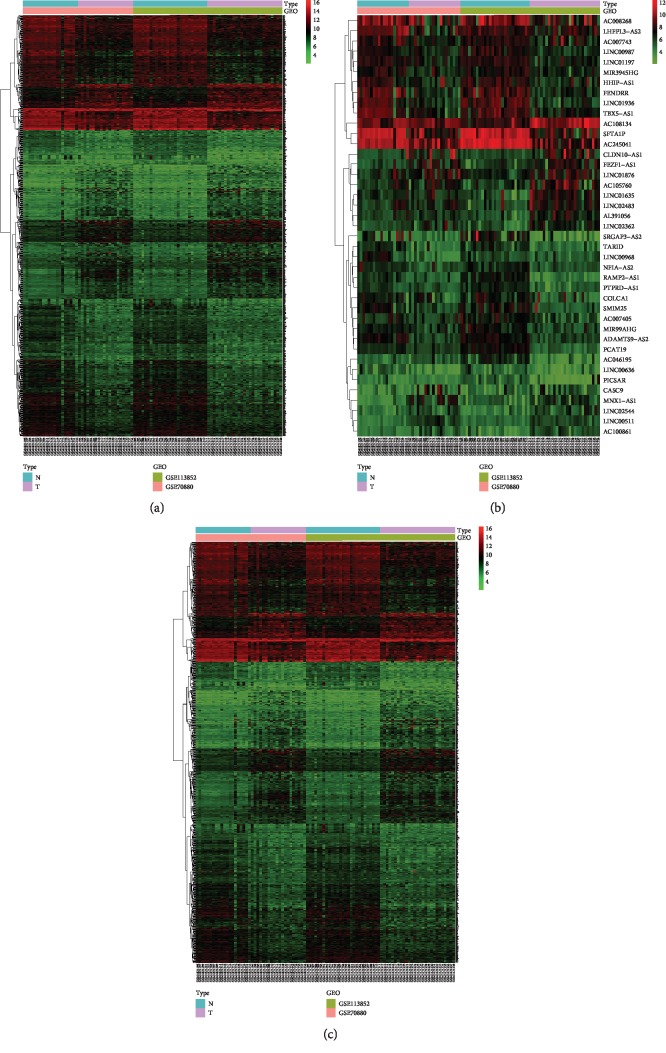
Heatmaps of differentially expressed genes in the merged dataset. (a) Heatmap of all differentially expressed genes. (b) Heatmap of differentially expressed lncRNAs. (c) Heatmap of differentially expressed mRNAs. The color represents the level of gene expression. From green to black to red, the levels of gene expression are increasing. lncRNA: long noncoding RNA; mRNA: messenger RNA; N: normal tissues; T: tumor tissues.

There were two datasets and a total of 47 lung cancer tissue samples and 47 normal tissue samples in this study (Table 1). We merged the two datasets into one dataset and then batch-normalized it to reduce variability. 846 genes (321 genes were upregulated and 525 genes were downregulated) were confirmed as DEGs. We divided the DEGs into mRNA group (a total of 805 mRNAs, 307 mRNAs were upregulated and 498 mRNAs were downregulated) and lncRNA group (a total of 41 lncRNAs, 14 lncRNAs were upregulated and 27 lncRNAs were downregulated) (Table 2). Volcano plot showed the results of gene differential analysis (Figure 1). Heatmaps showed gene expression changes (Figure 2).

### 3.2. ceRNA Network

**Figure 3:**
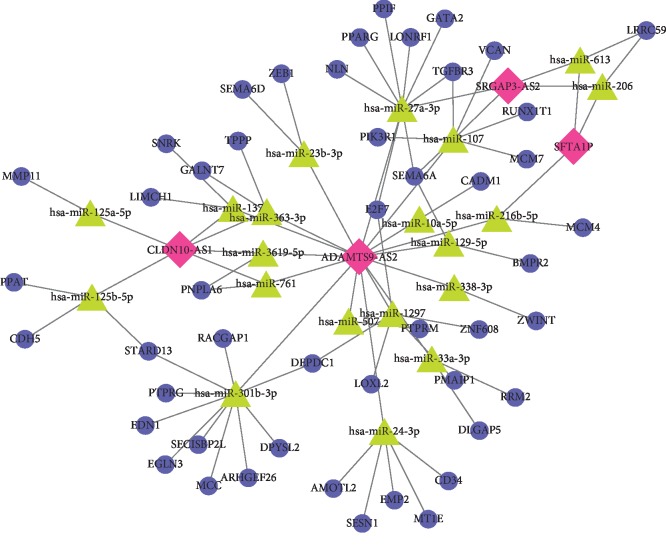
Competing endogenous RNA (ceRNA) network. Red diamond represents lncRNAs, green triangle represents miRNAs, and blue circle represents mRNAs. lncRNA: long noncoding RNA; miRNA: micro-RNA; mRNA: messenger RNA.

A total of 4 lncRNAs (CLDN10-AS1, SFTA1P, SRGAP3-AS2, and ADAMTS9-AS2), 21 miRNAs, and 48 mRNAs were involved in the ceRNA network (Figure 3). In tumor tissues, CLDN10-AS1 was upregulated, while SFTA1P, SRGAP3-AS2, and ADAMTS9-AS2 were downregulated. The number of nodes and lines in the network was 73 and 85, respectively. Among the 4 lncRNAs, the number of miRNAs that connected with ADAMTS9-AS2 was larger than that of other lncRNAs, which indicated the important role of ADAMTS9-AS2. miRNAs miR-125a-5p and miR-125b-5p were only connected with lncRNA CLDN10-AS1, which showed the special role of CLDN10-AS1 among the four lncRNAs.

### 3.3. GO and KEGG Pathway Analysis

**Figure 4:**
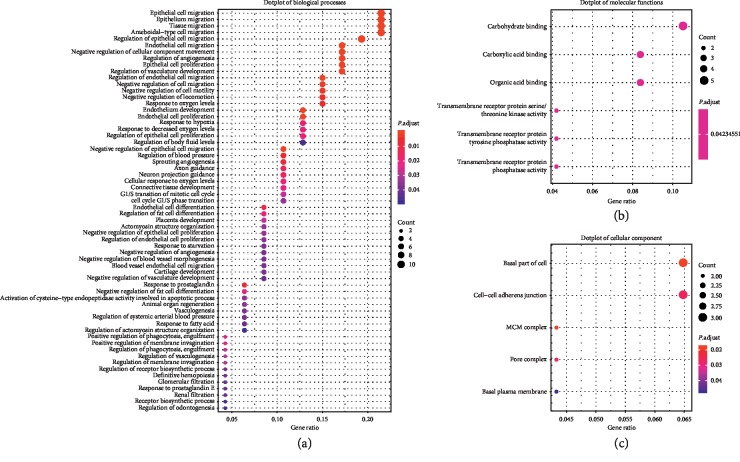
Dotplots of gene ontology (GO) analysis. (a) Dotplot of biological processes. (b) Dotplot of molecular functions. (c) Dotplot of cellular component.

**Figure 5:**
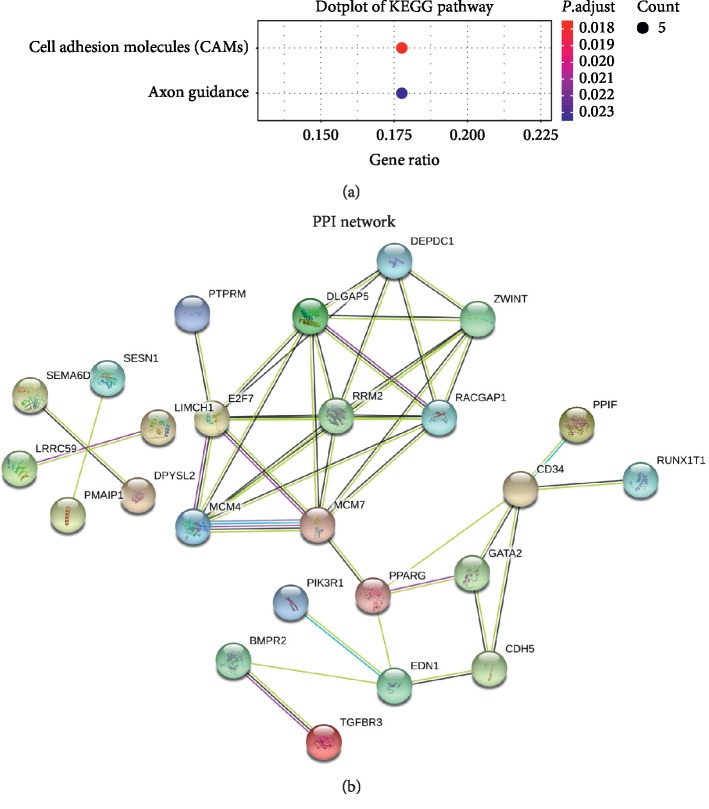
Dotplot of Kyoto Encyclopedia of Genes and Genomes (KEGG) pathway analysis and protein‐protein interaction (PPI) network. (a) Dotplot of KEGG pathway analysis. (b) PPI network.

GO terms and KEGG pathway analyses were performed by package clusterProfiler in software R. The results showed that biological processes were mainly associated with epithelial cell migration, regulation of epithelial cell migration, ameboidal-type cell migration, endothelial cell migration, endothelium development, and so on, while molecular functions were associated with carbohydrate binding, transmembrane receptor protein serine/threonine kinase activity, transmembrane receptor protein tyrosine phosphatase activity, transmembrane receptor protein phosphatase activity, carboxylic acid binding, and organic acid binding. Cellular component gathered in the basal part of cell, MCM complex, pore complex, cell-cell adherens junction, and basal plasma membrane (Figure 4). KEGG pathway analysis revealed the genes were only enriched in cell adhesion molecules (CAMs) and axon guidance (Figure 5(a)).

### 3.4. Protein-Protein Interaction

**Figure 6:**
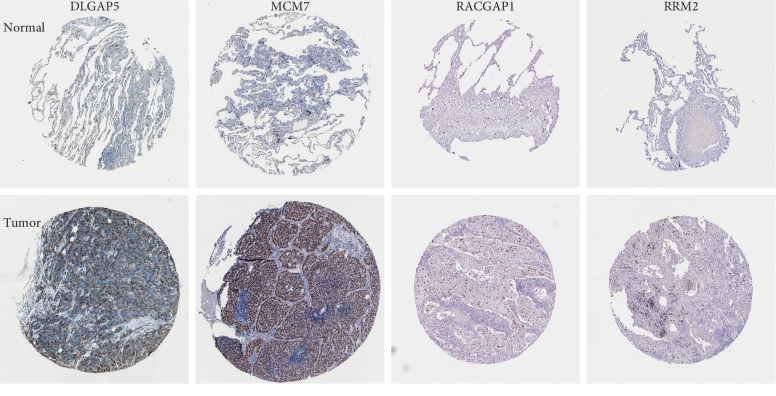
Immunohistochemistry (IHC) of DLGAP5, MCM7, RACGAP1, and RRM2 expression in lung adenocarcinoma (LUAD) and paired with normal tissue based on The Human Protein Atlas (THPA). DLGAP5, MCM7, RACGAP1, and RRM2 were upregulated in LUAD.

We uploaded the mRNAs in the ceRNA network to STRING to construct a PPI network (Figure 5(b)). There were a total of 25 nodes and 42 edges in the network. PPI enrichment *P* value was 1.07*e* − 07. We calculated the number of genes connected to each gene in the PPI network. Results revealed that five genes had the highest connectivity (DLGAP5, E2F7, MCM7, RACGAP1, and RRM2, and the number of genes connected with each of the five genes was seven). All of them were upregulated. Immunohistochemistry (IHC) in The Human Protein Atlas (THPA) (https://www.proteinatlas.org/) database demonstrated that DLGAP5, MCM7, RACGAP1, and RRM2 were upregulated in LUAD (Figure 6).

### 3.5. Survival Analyses in LUAD and LUSC

**Figure 7:**
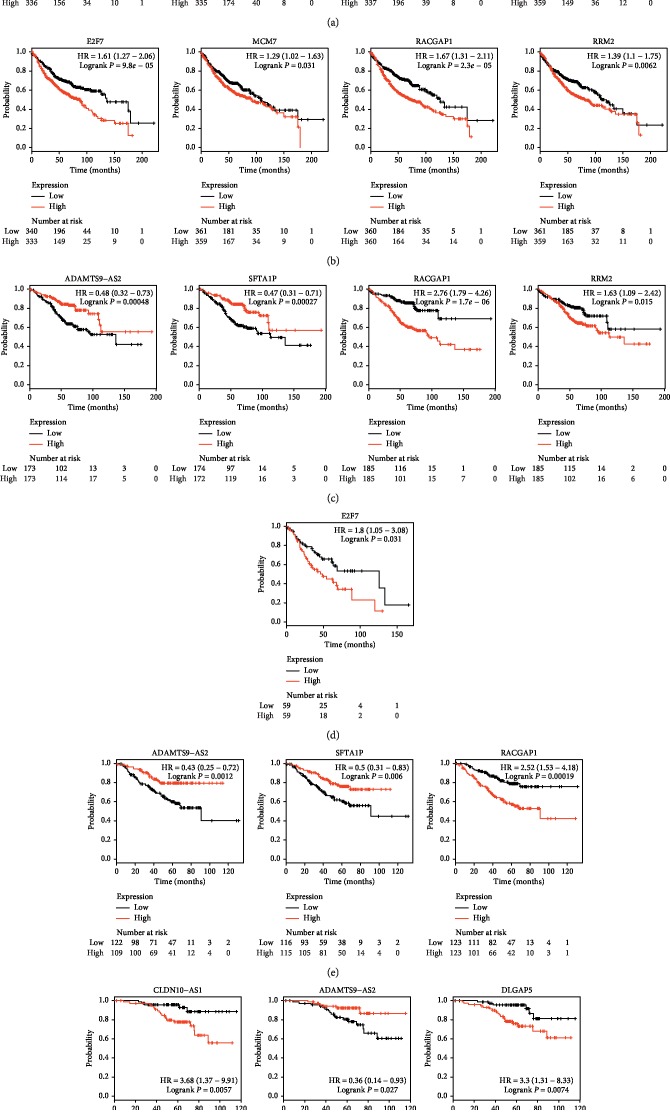
Kaplan–Meier (KM) analysis results of three lncRNAs and five mRNAs in cohorts of (a, b) LUAD patients, (c) stage I LUAD patients, (d) stage II LUAD patients, (e) LUAD patients with smoking history, and (f) LUAD patients without smoking history. lncRNA: long noncoding RNA. LUAD: lung adenocarcinoma.

**Figure 8:**
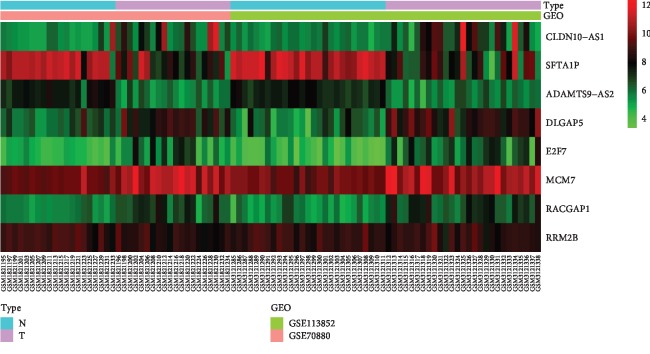
The heatmap showed the expression of the three lncRNAs and five mRNAs in the merged dataset. lncRNA: long noncoding RNA. N: normal tissues. T: tumor tissues.

We uploaded the four lncRNAs and five mRNAs to the Kaplan–Meier Plotter (http://kmplot.com/analysis/) to perform survival analyses in LUAD and LUSC, respectively (Figures 7(a) and 7(b)). The results showed that lncRNAs CLDN10-AS1 (logrank *P*=0.026), SFTA1P (logrank *P*=0.00071), and ADAMTS9-AS2 (logrank *P*=0.0082) were associated with prognosis of LUAD. Upregulation of CLDN10-AS1 predicted poor prognosis, while upregulation of the SFTA1P and ADAMTS9-AS2 was related to good prognosis. mRNAs DLGAP5 (logrank *P*=4.7*e* − 05), E2F7 (logrank *P*=9.8*e* − 05), MCM7 (logrank *P*=0.031), RACGAP1 (logrank *P*=2.3*e* − 05), and RRM2 (logrank *P*=0.0062) were associated with prognosis of LUAD. Their high expression related to poor prognosis. No lncRNAs and mRNAs were associated with the prognosis of LUSC. DLGAP5, E2F7, MCM7, RACGAP1, and RRM2 were considered as hub genes. The heatmap showed the expression changes of the three lncRNAs and five mRNAs in the merged dataset (Figure 8).

Furthermore, we performed survival analyses of the three lncRNAs and five mRNAs in the cohorts of stage I LUAD patients, stage II LUAD patients, LUAD patients with smoking history, and LUAD patients without smoking history. The results showed that lncRNAs SFTA1P (logrank *P*=0.00027) and ADAMTS9-AS2 (logrank *P*=0.00048) and mRNAs RACGAP1 (logrank *P*=1.7*e* − 06) and RRM2 (logrank *P*=0.015) were associated with the prognosis of stage I LUAD patients (Figure 7(c)); only mRNA E2F7 (logrank *P*=0.031) was associated with the prognosis of stage II LUAD patients (Figure 7(d)); lncRNAs SFTA1P (logrank *P*=0.006), ADAMTS9-AS2 (logrank *P*=0.0012), and mRNA RACGAP1 (logrank *P*=0.00019) were associated with the prognosis of LUAD patients with smoking history (Figure 7(e)); lncRNA CLDN10-AS1 (logrank *P*=0.0057), ADAMTS9-AS2 (logrank *P*=0.027), and mRNA DLGAP5 (logrank *P*=0.0074) were associated with the prognosis of LUAD patients without smoking history (Figure 7(f)).

### 3.6. Expression Levels and Diagnostic Values of Four lncRNAs in LUAD Confirmed through TCGA

**Figure 9:**
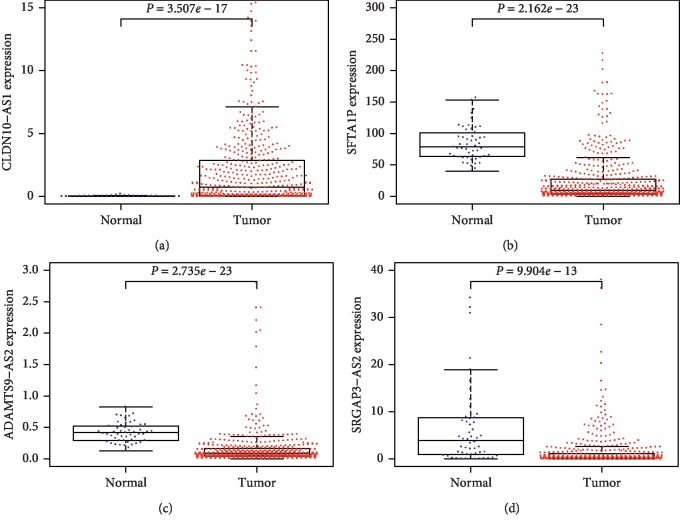
Expression levels of four lncRNAs in lung adenocarcinoma (LUAD) in TCGA. lncRNA: long noncoding RNA. TCGA: the cancer genome atlas.

**Figure 10:**
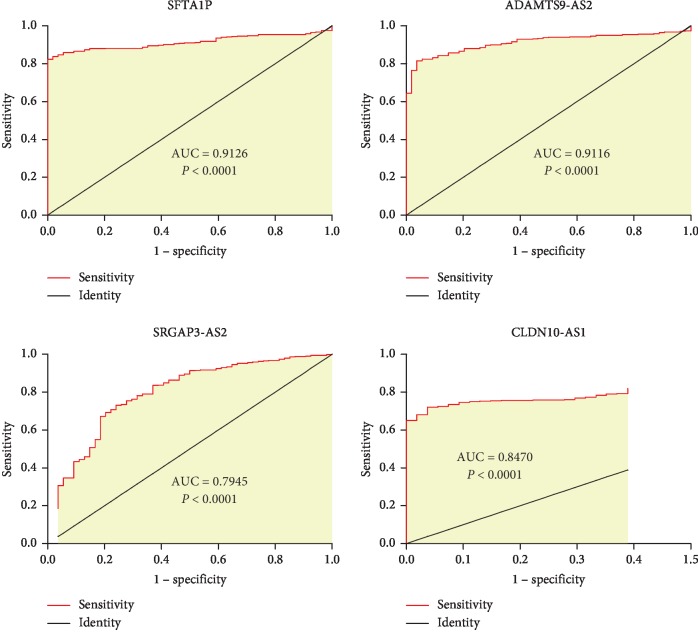
Receiver-operating characteristic (ROC) curves of four lncRNAs in lung adenocarcinoma (LUAD).

A total of 551 samples (54 normal samples and 497 tumor samples) of LUAD were downloaded from the TCGA database. After gene differential analysis, we observed that, in tumor samples, CLDN10-AS1 was upregulated, while SFTA1P, SRGAP3-AS2, and ADAMTS9-AS2 were downregulated (Figure 9). The results were consistent with our previous conclusions. Furthermore, the ROC curves showed that all of the four lncRNAs had diagnostic values in LUAD (Table 3, Figure 10).

## 4. Discussion

In the present study, we investigated the gene expression patterns of lncRNAs and mRNAs in lung cancer cells and corresponding adjacent tissues or normal tissues. We found that 525 genes were downregulated and 321 genes were upregulated in lung cancer cells. The ceRNA network revealed the correlation among lncRNAs, miRNAs, and mRNAs. Four lncRNAs (CLDN10-AS1, SFTA1P, SRGAP3-AS2, and ADAMTS9-AS2) were involved in ceRNA network and survival analyses showed that CLDN10-AS1, SFTA1P, and ADAMTS9-AS2 were associated with prognosis of LUAD. The PPI network showed the interaction among these mRNAs in ceRNA network and five mRNAs (DLGAP5, E2F7, MCM7, RACGAP1, and RRM2) had the highest connectivity. Survival analyses also revealed the relationship between the five mRNAs and the prognosis of LUAD, and they were confirmed as hub genes of LUAD finally. However, survival analyses indicated that these lncRNAs and mRNAs were not related to the prognosis of LUSC. To increase the credibility of our results, we verified the expression levels and measured the prognostic values of the four lncRNAs in LUAD through TCGA database. The results confirmed our conclusions and revealed that all the four lncRNAs were associated with the diagnosis of LUAD. lncRNAs CLDN10-AS1, SFTA1P, and ADAMTS9-AS2, and their related mRNAs DLGAP5, E2F7, MCM7, RACGAP1, and RRM2 might be biomarkers of LUAD.

Previous studies have explored the functions of lncRNAs as diagnostic and prognostic biomarkers [6]. HOTAIR is a famous cancer-related lncRNA and it is highly expressed in NSCLC and SCLC [10]. MALAT1 is another important lncRNA, and in patients with non-small-cell lung cancer, it is significantly related to metastasis potential and poor prognosis [19, 20]. Schmidt et al. indicated that MALAT1 could be considered as an independent prognostic parameter for both LUAD and LUSC [19, 20, 22]. Due to the high expression in LUAD, CCAT2 is a potential diagnostic biomarker for LUAD [22]. lncRNAs have also been studied as potential drug targets [6]. HOTAIR might be a therapeutic target in NSCLC because of its role in the chemoresistance to cisplatin [6]. Liu et al. indicated that MEG3 might be a potential therapeutic target in lung cancer, for tumor cells would be sensitive to cisplatin when MEG3 was overexpressed in A549 cells [23].

lncRNA SFTA1P had been reported previously. Huang et al. demonstrated that SFTA1P and CASC2 were associated with the regulation and development of LUSC and could be used as prognostic and predictive indicators of LUSC via integrated bioinformatics analysis [24]. Zhang et al. reported that the downregulation of SFTA1P affected LUAD patients' survival time but had no influence on LUSC patients. SFTA1P exerted tumor inhibition in LUAD [25]. There were no experiments on the mechanism of SFTA1P in lung cancer so far. However, this opens up many avenues of study to pursue on this topic.

The number of miRNAs that connected with lncRNA ADAMTS9-AS2 was the largest among the four lncRNAs, suggesting it was a critical lncRNA. Studies about lncRNA ADAMTS9-AS2 in lung cancer were rare. Liu et al. demonstrated that lncRNA ADAMTS9‐AS2 was lowly expressed in lung cancer tissues by qRT-PCR. In their study, high expression of lncRNA ADAMTS9-AS2 reduced proliferation ability and inhibited migration and elevated their apoptosis rate. They also verified the relationship among lncRNA ADAMTS9-AS2, mRNA TGFBR3, and miRNA miR-223-3p. ADAMTS9-AS2 increased TGFBR3 expression, but miR-223-3p decreased both of them. miR-223-3p targeted TGFBR3 to enhance the ability of proliferation, migration, and invasion of lung cancer. They came to a conclusion that DAMTS9-AS2, TGFBR3, and miR-223-3p might provide potential therapeutic targets in lung cancer [26].

miRNAs miR-125a-5p and miR-125b-5p only connected with lncRNA CLDN10-AS1, indicating lncRNA CLDN10-AS1 had a different binding trend among the four lncRNAs. CLDN10-AS1 might play a role in the development and progression of lung cancer, by regulating the G1/S transition of mitotic cell cycle through miR-125b-5p/PPAT and by regulating endothelium development, angiogenesis, and cell-cell adherens junction through miR-125b-5p/CDH5, respectively. Furthermore, CLDN10-AS1 was associated with the prognosis of LUAD (logrank *P*=0.026), indicating the potential functions in the prognosis of LUAD patients. It is noteworthy that CLDN10-AS1 has not been reported in lung cancer. For the first time, we confirmed the crucial role of lncRNA CLDN10-AS1 in LUAD. It might be related to the diagnosis, ability of migration, and prognosis of LUAD.

Limitations existed in our study. (1) We only included two datasets. Although there were some other datasets, their sample size was too small. In order to increase the quality of our study, we excluded datasets with a sample size less than 40. (2) The two datasets are noncoding mRNA and lncRNA microarray, so we used the probe reannotation method to annotate gene symbol, which might drop some genes due to the failure of matching the probes. (3) Neither dataset contained clinical information. We could only perform survival analyses through the Kaplan–Meier Plotter (http://kmplot.com/analysis/). (4) We did not validate our results by experiment, giving us a direction for future research.

## 5. Conclusion

In our study, we identified the differentially expressed lncRNAs CLDN10-AS1, SFTA1P, SRGAP3-AS2, and ADAMTS9-AS2 by analyzing gene expression profiles from GEO. Among them, CLDN10-AS1, SFTA1P, and ADAMTS9-AS2 and their related mRNAs DLGAP5, E2F7, MCM7, RACGAP1, and RRM2 were associated with the prognosis of LUAD, suggesting they were more critical in LUAD. What is more, the four lncRNAs had diagnostic values in LUAD. lncRNAs CLDN10-AS1, SFTA1P, and ADAMTS9-AS2 and their related mRNAs DLGAP5, E2F7, MCM7, RACGAP1, and RRM2 might be biomarkers of LUAD. For the first time, we confirmed the important role of lncRNA CLDN10-AS1 in LUAD. It might be related to the diagnosis, ability of migration, and prognosis of LUAD.

## Figures and Tables

**Table 1:** Details of datasets from Gene Expression Omnibus database.

Series accession	Platform	Tumor	Normal	Contributors
GSE70880	GPL19748	20	20	Yuan J., Yue H, Luo J., Chen R.
GSE113852	GPL16847	27	27	Polycarpou-Schwarz M., Roth A, et al.

**Table 2:** Differentially expressed lncRNAs and mRNAs of merged dataset.

lncRNAs	Upregulated	MNX1-AS1, FEZF1-AS1, CLDN10-AS1, LINC00511, LINC01635, LINC02544, LINC02362, AC105760, AC100861, LINC02483, AC108134, AL391056, CASC9, LINC01876
	Downregulated	PICSAR, AC007743, MIR3945HG, MIR99AHG, AC007405, NFIA-AS2, LINC00987, AC046195, LINC00968, COLCA1, LINC00636, PTPRD-AS1, LINC01197, HHIP-AS1, LHFPL3-AS2, SMIM25, PCAT19, ADAMTS9-AS2, TBX5-AS1, TARID, AC008268, RAMP2-AS1, FENDRR, AC245041, SRGAP3-AS2, LINC01936, SFTA1P
mRNAs	Upregulated	FAM83A, MMP12, ANLN, TOP2A, CTHRC1, TPX2, CST2, ASPM, SLC2A1, KIF20A, UBE2C, CDCA2, KIF4A, KIF2C, DNAJC22, BUB1, PITX1, RRM2, CCNB2, CKAP2L, E2F8, MCM10, CDC20, KIF14, UHRF1, TTK, DLGAP5, CDCA7, PLPP2, SAPCD2, CP, LGSN, FOXM1, KRT80, CLSPN, MMP11, CDK1, CDKN3, TOX3, SCG5, SULF1, HMMR, NUF2, KIF11, CDC25C, NDC80, SLC22A18AS, SPP1, CHEK1, CENPA, GPT2, PRAME, TK1, GALNT14, CCNA2, IGFL2, CENPU, FAP, STIL, SLC44A5, ARHGEF39, MAP7D2, CCNO, ESPL1, COL3A1, RAD51AP1, SGPP2, CCNE1, GPX2, OCIAD2, SEMA4B, E2F7, KIF15, AUNIP, ARNTL2, SYT12, RAB26, EZH2, LMNB1, SPC24, ATP10B, AGMAT, PLK1, PLOD2, RECQL4, FAM72A, CDKN2A, FERMT1, EGLN3, SERINC2, AGR2, BRIP1, FGF11, TFAP2A, ZWINT, S100P, CENPF, PI15, MCM4, GALNT6, HELLS, MB, ORC6, CELSR3, LY6K, ATAD2, RALGPS2, CYP27B1, SLC2A5, STYK1, GTSE1, MMP13, RHOV, POLE2, RNF183, RHBDL2, ITPKA, TMEM45B, SGO2, ONECUT1, PRSS3, KNL1, KCNN4, GYG2, ABCC3, TIMELESS, ECT2, XRCC2, EPCAM, DSG2, JPT1, FANCI, GRHL1, DEPDC1, BPIFA2, CIT, FAM83B, GPC2, LOXL2, CEACAM1, PARPBP, GALNT7, CHGB, WDR62, FHL2, HIST1H2BE, CENPM, VWDE, TRIM31, AKR1B10, SFXN1, HILPDA, MYBL2, HIST1H2BI, FGL1, SLC16A14, MELTF, CXCL9, UGT8, AK4, STK32A, ADGRF1, NGEF, TFR2, KIF18A, HNF4G, PFKP, MZB1, UCHL1, ADAMTS16, RACGAP1, SLC17A9, CD19, P4HA3, ETV4, TNS4, TTYH3, SLFN13, STRA6, CARD14, CDCA8, CKS1B, FAM83D, CHI3L1, TNFRSF21, HIST1H2BC, FOXP3, CCDC34, FBXO32, GLYATL1, GSDMB, ESRP1, ARHGEF16, GAD1, SRPK1, TEX11, SLC7A5, HIST1H3H, TDRD5, FAM83F, FANCA, IL4I1, IGSF9, ESCO2, PPAT, HCN4, HIST1H2BG, KIF26B, MYO19, BICDL1, TDO2, CNTNAP5, BAIAP2L1, CPNE4, SLC12A8, LGI2, POC1A, LARGE2, RCC1, KIF18B, C12orf56, ZBED6CL, DBNDD1, CDC7, SYNJ2, NLN, FCN2, NECTIN4, LSR, CA12, MFAP2, UNC5CL, PTGES, BRCA1, PROM2, CGREF1, VTCN1, MTNR1A, SLC35F2, COL22A1, HNF4A, ERO1A, GSDMC, VCAN, PDCD2L, BZW2, KRTCAP3, AKR1B15, DTL, NCAPG2, SMKR1, PRSS1, SERPINB5, SHMT2, CPXM1, CHEK2, MCIDAS, EEF1AKMT4, GPR87, TONSL, GRHL2, SLC50A1, KCNQ5, CNTNAP2, C1orf53, BPIFB6, PRR19, PRR11, POSTN, KCNK1, MCM7, CIP2A, HIST3H2A, GAPDH, TUBB2B, BCO1, SUSD4, MTBP, LRRC59, STRIP2, PTHLH, PPIF, CLDN4, RNFT2, SYT7, SSR4, E2F1, VIL1, FCRL2, KIF22, BRDT, RNF43, TPD52, AOC1, JPT2, CDH1, DIRAS1, SCIN, TIGIT, PMAIP1, B3GNT5, MYEOV, VPREB3, P3H4, KLK6, BRI3BP, SLC39A11
	downregulated	SLC25A27, FAM184A, ZNF106, TJP1, TEKT2, PCYT1B, ITPRIP, EDIL3, GFOD1, SFTA3, TTLL10, AMIGO1, MT1E, SMIM10, LONRF1, PROK2, CFAP157, PODXLRSPO3, ELMO1, KLHDC1, SHROOM4, SMAD6, GPR146, SULT1A2, RASGRF1, NPR3, CD55, MACF1, TTLL11, MATN2, MRAS, ZNF608, PPP1R14C, RND3, IER2, DCN, PTPRG, TAL1, SLC2A3, FRAS1, PIFO, AMOTL2, MAP6, SFRP5, PLA1A, SLC27A3, ARHGAP20, RAB11FIP1, COBL, GNG7, TCF4, SLCO1A2, USP2, MAP7D3, SIRPB1, SORBS3, BMX, MYLK, DUOXA1, CNGA4, CACHD1, C1orf194, RASSF2, EFCAB12, SECISBP2L, SNRK, RGS22, SYNPO2, PTPRN2, ITGA10, NHSL1, HOXA4, CCL3, RAB11A, BMP6, PHLDB2, MYOCD, TSLP, MFSD2A, CADM1, GALNT16, VIM, MVB12B, TFPI, ADGRG6, CLEC12A, NXF3, ADPRH, ERG, GCOM1, KANK3, C1orf189, VSIR, SLC9A3R2, WNT11, HGF, LMO7, CES4A, PNPLA6, SPEF1, C1orf198, CST6, ABLIM1, PRICKLE2, ID2, PKNOX2, RPS6KA2, EFHB, SLC18A2, APOLD1, SPI1, ACACB, ID3, ADAMTS15, ECM2, SYNDIG1L, ELF5, DCDC2, HYAL2, HACD4, CD300LG, RBP7, TLL1, FAT4, BMPR2, GMFG, TMEM130, CCDC68, SIGLEC6, DRC3, SOX5, ARAP3, TSPAN19, JPH2, SYDE2, MYRIP, THSD4, CSRP1, FILIP1, VWA3A, LAMA4, SCTR, SPATA4, LBH, PTPN13, LAMA3, DISP1, HMGCLL1, ABHD6, STARD9, DENND3, NCALD, WNT2B, NFASC, MAPK4, DTHD1, ESYT3, NEXN, DNAJB4, TMEM139, ACOXL, ST6GALNAC3, MAP2, MCC, ATOH8, AKAP12, NEBL, CAV1, ZEB1, CLDN5, EPB41L2, RECK, TRPC6, TTLL7, SERTAD1, ANGPT1, SLC14A1, PYGM, CYB5A, IGSF22, DOCK4, SESN1, DGKG, SELENBP1, TUBB6, C1orf162, NLRC4, MSR1, NKD2, ALDH1A2, MEIS1, ETS2, PHACTR1, PRDM6, TSPAN18, AC023509, ADCY4, STAC, CDH19, JCAD, SERPINA1, SOBP, FLRT3, MAL, COLEC12, PIK3R1, IQCN, FEZ1, CASS4, NAPSA, NAV3, PLXNA2, NDRG4, CLDN18, RGS9, MSLN, FGR, EML1, RASGEF1B, PLCE1, RBMS2, NIM1K, SH3D19, METTL7A, RUNX1T1, LRRN4, PIGR, CALCOCO1, C14orf132, KL, SCEL, C16orf89, CFAP43, PDZRN3, FRMD3, CTSG, KLF6, GATA2, NR4A1, FGFR2, GIMAP1, CRIM1, C9orf24, FLI1, MGP, CXCL12, MFAP5, DNAAF1, PGM5, AATK, WFS1, SGIP1, HYAL1, SOX13, WNT7A, STARD13, NFIX, KCNK17, MDH1B, ROS1, ADGRE1, GATA6, DMD, ART4, USHBP1, SEMA6A, CA1, ADGRL2, CTXND1, TACC1, FAT3, SEMA6D, CPED1, ABCG2, IL7R, CD34, ARHGAP6, HECW2, ITLN1, ENG, DKK2, PRKG1, LRRC36, CHST9, SEMA3D, CFAP221, GADD45B, PTGFR, IRX2, PLCL1, SH3GL2, CAVIN1, SORBS1, SEMA3B, DNAI2, ARHGEF26, RADIL, GLIPR2, NKD1, SCN4B, RAI2, CHIA, PIP5K1B, RSPH9, SYNE1, RASIP1, TTN, SELENOP, ABCA6, TEX14, RAPGEF4, PECAM1, SLCO2A1, AKAP14, OTUD1, NTNG1, HEG1, MAB21L4, SPTBN1, RSPO2, ZBTB16, DPEP2, FBLN5, TPPP, SLC46A2, SASH1, PCDHGC3, LRRC32, CORO2B, TNS2, OLFML1, COL6A5, PALMD, CASKIN2, ADTRP, COL13A1, PDE5A, JAML, DPYSL2, NRN1, LMCD1, GYPC, PLCB4, CRTAC1, RANBP3L, P3H2, GUCY1A2, GPM6B, NDRG2, PTPRM, EDNRB, PGC, PRX, NEDD9, DENND2A, ESAM, PLEKHH2, LEPR, PDZRN4, LMO2, TMEM204, FBLN1, ODAM, MYH11, ADGRD1, THSD1, TMEM212, WASF3, MYRF, ATF3, MSRB3, ZNF366, VSIG4, FGD5, PTPRB, DLC1, TNS1, FGF10, HIF3A, C1QTNF7, RHOJ, CLEC1A, PPP1R15A, ATP13A4, CXCL2, KLF2, LAMC3, ANXA3, TIE1, SULT1C4, SUSD2, SELP, PPARG, CSRNP1, WFDC1, ITIH5, SFTPB, NOSTRIN, JAM2, SMTNL2, PKHD1L1, PID1, GRK5, KCNK3, PDZD2, DUSP1, LRRK2, LIMCH1, PCOLCE2, LDB2, GPRC5A, IL1RL1, FAM13C, SVEP1, AFF3, HLF, RP1, NCKAP5, ERICH3, DUOX1, EMP2, EPAS1, CCDC141, PPP1R14A, SLIT2, CLEC14A, MMRN1, PDK4, SLC39A8, EDN1, ADAMTS1, VSIG2, COX4I2, ABCA8, GRASP, ABCA3, MYCT1, ROBO2, CALCRL, ANXA8, ANKRD29, CDH5, LIMS2, TEKT1, ZNF385B, NECAB1, ROBO4, SLIT3, ADAMTSL3, PLA2G4F, VIPR1, LTBP4, GPC3, KLF4, C11orf88, CACNA2D2, CNTFR, VEPH1, ADAMTS8, PI16, FGFR4, RAMP3, RGCC, ABI3BP, HHIP, CA4, TGFBR3, CLIC5, RXFP1, CCL14, COL6A6, CYP4B1, SCARA5, TPPP3, ACKR1, AOC3, ACTN2, GRIA1, MFAP4, SCUBE1, STXBP6, DNASE1L3, GPM6A, CLEC3B, RTKN2, TNXB, LGI3, MAMDC2, LYVE1, BTNL9, FOSB, GKN2, FCN3, DES, FHL1, FAM107A, SFTPC

**Table 3:** Diagnostic values of the four lncRNAs.

lncRNA	Cutoff value	AUC (95% CI)	Sensitivity (%)	Specificity (%)
SFTAP1	39.89	0.9126 (0.886–0.935)	82.29	100.00
ADAMTS9-AS2	0.212	0.9116 (0.885–0.934)	81.49	96.30
SRGAP3-AS2	0.921	0.7945 (0.758–0.828)	73.04	75.93
CLDN10-AS1	0.078	0.847 (0.814–0.876)	72.03	96.30

AUC: area under the curve; CI: confidence interval. Cutoff value is the best expression of lncRNA for the diagnosis of lung adenocarcinoma.

## Data Availability

All data can be obtained from GEO (https://www.ncbi.nlm.nih.gov/gds/), TCGA (https://portal.gdc.cancer.gov/), and THPA (https://www.proteinatlas.org/).
